# Possibility of persistent current in S-states

**DOI:** 10.1038/s41598-024-63838-7

**Published:** 2024-06-07

**Authors:** Chanchal Yadav, Brijender Dahiya, Vinod Prasad

**Affiliations:** 1 Department of Physics and Astrophysics, University of Delhi, Delhi, 110007 India; 2 Department of Physics, Hansraj College, University of Delhi, Delhi, 110007 India; 3 Department of Physics, Swami Shraddhanand College, University of Delhi, Delhi, 110036 India

**Keywords:** PTDRSC potential, Double ring-shaped potential, Persistent charge current, Induced magnetic field, Physics, Atomic and molecular physics

## Abstract

In this study, we investigate the profound impact of the Pöschl–Teller double-ring-shaped Coulomb (PTDRSC) potential to induce persistent currents within the S-states of the hydrogenic atom. The confinement of the system is achieved through an impenetrable spherical boundary. Leveraging first-order perturbation theory, we quantify the charge current across various states induced by the PTDRSC potential with its inherent angular and azimuthal dependence, leading to angular and azimuthal distortion, respectively. Notably, persistent currents are observed within S-states without external excitation mechanisms. The magnitude of the induced current is intricately linked to the strength of the PTDRSC potential parameters. These results underscore the prospect of manipulating persistent currents and their associated induced magnetic fields within S-states by tailoring the potential strength and confining boundary size. This discovery presents a compelling avenue for the controlled generation and experimental verification of induced S-state magnetism, opening new possibilities for innovative applications.

## Introduction

Persistent currents in quantum systems are a manifestation of the quantization of angular momentum and energy levels. Discrete energy levels can lead to quantized persistent currents without an applied voltage. Persistent currents can arise in non-superconducting systems when subjected to magnetic fields. Levy et al.^1^ demonstrated the presence of these currents at the mesoscopic level. In 1987, D. F. Brewer^2^ experimentally observed persistent currents in superfluid Helium. Since then, it has been theoretically explored in a number of quantum systems^3–6^. For example, in metallic rings^3^, semiconducting 1-D rings^4^ and various nano structures^5^. Peeks et al.^5^ showed the existence of ring currents around the perimeters of molecular nanorings. The direction of persistent currents in such nanorings depends on the number of electrons. They have shown that the experimental results match quite surprisingly with the theoretically calculated currents^6–8^. For a recent review^9^. Recently, structured laser beams i.e., laser beams that carry orbital angular momentum (OAM), have been used to generate persistent currents in atomic, molecular, and other quantum systems. For example, Koksal et al.^10^ demonstrated that a linearly polarised Gaussian beam can induce a persistent current of a sizeable amount if the OAM of the beam is greater than zero. However, their study shows that currents are in the nA range. Theoretical studies on generating charge currents in complex molecules are also reported^11–14^. During the last decade, the much sought persistent current was experimentally measured indirectly^3^.

Persistent currents are responsible for generating magnetic fields due to the circulating current in a superconducting loop. This effect is used in various applications, such as to create a strong magnetic field in superconducting magnets used in MRI machines, fusion reactors and particle accelerators. Also, these currents can generate magnetic fields that oppose the external magnetic field. Bandrauk and his coworkers have shown that circularly polarized laser pulses can induce large persistent electronic currents in molecular systems. In particular, they have focused on $H_2^+$ and $H_2$ molecular systems. They have shown that such generated persistent currents can induce magnetic fields, which are also time-dependent and are in the sub-picosecond range^15–17^. Recent advancements have allowed for control over supercurrent dynamics in fermionic systems. In 2022, Pace et al.^18^ demonstrated control over supercurrents in fermionic rings, achieving precise manipulation of persistent currents. Cai et al.^19^ also showed the potential of manipulating ultracold fermionic atoms, generating enduring currents in a ring with lifetimes over ten seconds. An innovative method for achieving electron nanobunching using a two-color laser pulse that interacts with a microstructured foil was proposed^20^. Their approach enabled the direct generation of ultra-intense attosecond pulses.

Spherical confinement can induce large charge currents in atomic systems^21^. Bahar^22^ theoretically calculated persistent charge currents and induced magnetic fields in different states of Li and Na atoms that are immersed in a quantum plasma environment and compressed within spherical confinement. In a spherically bound hydrogen atom, a unidirectional persistent current may be produced by an orientated molecule with circular polarisation^23^. Barth^24^ investigated the impact of short, intense, circularly polarized laser pulses on the persistent currents and induced magnetic fields of hydrogen atoms and hydrogenic ions by considering electron spin. The investigation about generating ultrafast magnetic fields in a molecular medium using circularly polarized attosecond ultraviolet laser pulses^25^ holds great importance for experimental research in various fields. Prasad et al.^26^ examined the effect of an external magnetic field and a laser pulse on the charge currents and induced magnetic field in a spherically enclosed hydrogen atom under the influence of non-central potential. Their results demonstrated the effects of the external magnetic field and the laser pulse on these properties. They further investigated the impact of an ultrafast polarized laser pulse on these properties for the same system^27^, which revealed the effectiveness of the laser pulse in comparison to the effects of the non-central potential parameters. Talwar et al.^28^ investigated the charge currents and induced magnetic field of a 2D hydrogen atom when subjected to external electric and magnetic fields. The study provided insights into current and magnetic field patterns for various states. In 2021, Bahar^29^ conducted a theoretical study exploring the behaviour of charge currents and induced magnetizations in a hydrogen atom when subjected to the plasma-modified Hartmann potential. The research delved into the analysis of plasma shielding, spherical confinement, and noncentral effects of the generation and manipulation of charge currents and induced magnetic fields. Recently, Lumb et al.^30^ observed the adiabatic magnetic pulses while investigating the behaviour of a 2D quantum disk under the influence of a non-central potential when exposed to a linearly polarized laser pulse along with a static electric field.

Radial potentials fail to explain certain phenomena, such as the presence of ring-shaped molecules^31^ or distorted nuclei^32^. In such scenarios, PTDRSC potential emerges as a promising alternative. The PTDRSC potential refers to a combination of Coulomb potential, Pöschl-Teller potential and a double-ring-shaped inverse square potential. The asymptotic expression of the PTDRSC potential is identical to that of the Coulomb potential^33^. In 2015, Chabab et al.^34^ gave analytical solutions of the Schrödinger equation for a class of non-central physical potentials (including PTDRSC potential) where they derived energy eigenvalues and eigenfunctions in the position-dependent effective mass framework. The time-dependent Schrödinger equation is solved to study the time evolution of a system under the Pöschl-Teller double-ring shaped harmonic potential^35^. In a recent investigation, Nguyen^36^ estimated the impact of the two asymmetry parameters inherent in the Pöschl-Teller quantum well confinement potential on its optical characteristics. Their research highlighted the Pöschl-Teller potential as a favourable option for applications in optoelectronic devices. Lin and Yuan^37^ analytically solved the Schrödinger equation with the PTDRSC potential to obtain solutions for bound states. They expressed the normalized angular wave function using Jacobi polynomials and the normalized radial wave function using Laguerre polynomials. Hassanabadi et al.^38^ obtained an analytical solution to the Klein-Gordon equation for equal scalar and vector PTDRSC potentials having applications in quantum chemistry and nuclear physics. The PTDRSC potential can affect the energy levels and wavefunctions of the particles, providing insights into their behaviour and properties. Additionally, the potential can be used to model interactions between particles in nuclear physics, helping to understand phenomena such as nuclear reactions and decay processes.

All these investigations mentioned above inspired us to explore the impact of PTDRSC plasma potential. Inspired by the analytical work of Chabab et al.^34^, this study explores a spherically enclosed hydrogenic atom confined in PTDRSC potential. The hydrogenic atom has been chosen as the focus of this study to understand the effect of *Z*. This investigation focuses on the energy spectrum estimation along with persistent currents and induced magnetic fields by varying the PTDRSC potential parameters and size of the spherical boundary. The structure of the present work is as follows: The solution of the time-independent Schrödinger equation using the separation of variables with PTDRSC potential in spherical coordinates is elaborated in the methods section. The results section explains the effect of PTDRSC potential parameters and spherical boundary size on persistent currents and their corresponding induced magnetic fields in different states. Lastly, the conclusion section summarizes the main outcome of our findings. Atomic units have been used in this work, i.e., $\hbar =m_{e}=e=1.$

## Methods

Consider a spherically enclosed atom under the influence of Pöschl-Teller double-ring-shaped Coulomb (PTDRSC) potential, characterized by the following form:

1 $$\begin{aligned} V_P(r,\theta , \phi )&= -\frac{b}{r}+\frac{1}{r^{2}}\left[ \frac{\beta }{\sin ^{2}\theta }+ \frac{a(a-1)}{\cos ^{2}\theta } \right] + \frac{1}{r^{2}\sin ^{2}\theta } {\left[ \frac{\alpha ^2 c(c-1)}{\cos ^2\alpha \phi }+\frac{\alpha ^2 d(d-1)}{\sin ^2\alpha \phi }\right] } \end{aligned}$$

here *a*, *c* and $$d > 1$$, $$b > 0$$, $$\alpha $$, and $$\beta $$ are parameters of the potential. When $$a = c = d = 1$$ and $$\beta = 0$$, PTDRSC potential simplifies to the Coulomb potential, which holds significant importance in quantum physics. Furthermore, when $$c = d = 1$$, PTDRSC potential reduces to the double ring-shaped Coulomb potential. For the present analysis, we constrain *b* to *Z*, where Z represents the atomic number of the hydrogenic atom. Z=1 for the hydrogen atom (H), Z=2 for the helium-ion (He$$^{+1})$$, and Z=3 for the lithium-ion ( Li$$^{+2})$$.

The time-independent Schrödinger equation for this system expressed in a spherical polar coordinate system is:

2 $$\begin{aligned} \left[ -\frac{1}{2}\left( \frac{1}{r^2}\frac{\partial }{\partial r}\left( r^2\frac{\partial }{\partial r} \right) +\frac{1}{r^2 \sin \theta }\frac{\partial }{\partial \theta }\left( \sin \theta \frac{\partial }{\partial \theta } \right) +\frac{1}{r^2 \sin ^2\theta }\frac{\partial ^2}{\partial \phi ^2}\right) +V_{P}(r,\theta , \phi )+V_{c}(r)\right] \ \psi _{nlm}(\textbf{r})&= E\ \psi _{nlm}(\textbf{r}) \end{aligned}$$

where $V_{c}(r)$ represents the enclosing boundary potential, mathematically defined as:

3 $$\begin{aligned} V_{c}(r)&= {\left\{ \begin{array}{ll}0 &{} \text {when } r< r_{0} \\ \infty &{} \text {when }r\ge r_{0}\end{array}\right. } \end{aligned}$$

The PTDRSC potential has the following form:

4 $$\begin{aligned} V(r,\theta , \phi )&= V_1(r)+\frac{1}{r^{2}}V_2(\theta ) +\frac{1}{r^{2}\sin ^{2}\theta }V_3(\phi ) \end{aligned}$$

so, the wave function for the system can be decomposed into radial, angular part, and azimuthal components, following standard notation:

5 $$\begin{aligned} \Psi _{nl'm'}(\textbf{r})&= R_{nl'}(r) \Upsilon _{l'm'}(\theta ,\phi ) = \frac{U_{nl'}(r)}{r}\Theta _{l'm'}(\theta )\Phi _{m'}(\phi ) \end{aligned}$$

Upon substituting the assumed form of this wave function into Eq. (2), we obtain a set of second-order differential equations of the following form:

6 $$\begin{aligned} \left[ -\frac{1}{2}\frac{d^{2}}{dr^{2}}+\frac{L^2}{2r^{2}}-\frac{Z}{r}\right] U_{nl'}(r)&= E_{n'l'} \ U_{nl'}(r) \end{aligned}$$

7 $$\begin{aligned}&\left[ -\frac{d^2}{d\theta ^2}-\cot \theta \frac{d}{d\theta }+\frac{\Lambda ^2}{\sin ^{2}\theta }+2\left( \frac{\beta }{\sin ^{2}\theta }+ \frac{a(a-1)}{\cos ^{2}\theta }\right) \right] \Theta _{l'm'}(\theta )&= L^2 \ \Theta _{l'm'}(\theta ) \end{aligned}$$

8 $$\begin{aligned}&\left[ -\frac{d^2}{d\phi ^{2}}+2\gamma \alpha ^2{\left( \frac{c(c-1)}{\cos ^2\alpha \phi }+\frac{d(d-1)}{\sin ^2\alpha \phi }\right) }\right] \Phi _{m'}(\phi )&= \Lambda ^2 \ \Phi _{m'}(\phi ) \end{aligned}$$

here the separation constants $L^2$ and $\Lambda ^2$ have been introduced. First of all, we solve Eq. (8) to find azimuthal wave functions, $\Phi _{m'}(\phi )$ and eigenvalues, $\Lambda ^2$ which are then substituted in Eq. (7) to obtain angular wave functions, $\Theta _{l'm'}(\theta )$ and eigenvalues, $L^2$. These values of $L^2$ are used in Eq. (6) to determine energy eigenvalues, $E_{nl'}$ and radial wave functions, $U_{nl'}(r)$. Thus, the complete wave function can be estimated using Eq. (5).

The probability current density is a vector field providing insights into the spatial distribution of the probability of finding a particle and its motion. The probability current density for the system in state $\Psi _{k}$ (*k* is used to denote different combinations of $n, l',$ and $m'$) is given by:

9 $$\begin{aligned} \textbf{J}_{k}(\textbf{r})&= \frac{i}{2}\left( \Psi _{k}(\textbf{r})\mathbf {\nabla }\Psi _{k}^*(\textbf{r})-\Psi _{k}^*(\textbf{r})\mathbf {\nabla }\Psi _{k}(\textbf{r})\right) \end{aligned}$$

Using the expression for $\mathbf {\nabla }$ in spherical polar coordinates, the radial (*r*) and angular ($\theta$) components of $\textbf{J}_k(\textbf{r})$, i.e., $\textbf{J}_k(r)$, and $\textbf{J}_k(\theta )$, cancel out resulting in only an azimuthal ($\phi$) component of current density, i.e., $\textbf{J}_k(\phi )$, which can be evaluated. The persistent current associated with the state $\Psi _k$ is given by

10 $$\begin{aligned} I_{k}&= -\int \int \textbf{ J}_{k}(\phi ).\textbf{ds} \end{aligned}$$

11 $$\begin{aligned} \textbf{ ds}&= r\ dr\ d\theta \ \hat{e}_{\phi } \end{aligned}$$

12 $$\begin{aligned} I_{k}&= -\int _{0}^{\pi } \int _{0}^{r_0} \textbf{ J}_{k}(\phi )\ r\ dr\ d\theta \ \hat{e}_{\phi } \end{aligned}$$

The magnetic field generated by this persistent current in a state is determined by applying the Biot-Savart law.

13 $$\begin{aligned} \vec {B}\left( \textbf{r}\right)&= -\frac{\mu _0}{4\pi } \int _{V'} \frac{\textbf{J}_{k}(\mathbf {r'})\ \text {x} \ (\textbf{r}-\mathbf {r'})}{|\textbf{r}-\mathbf {r'}|^3} dV' \end{aligned}$$

where $\mu _0$ represents the permeability of free space. The magnetic field generated at the nucleus ($\textbf{r}=0$) of the hydrogenic atom for any state, $\Psi _k$ is computed using Eq. (13).

## Results

Pöschl–Teller double-ring-shaped Coulomb (PTDRSC) potential is a non-spherically symmetric potential having azimuthal dependence that adds further interest to the current investigation. A spherically confined system refers to a system enclosed by an impenetrable spherical boundary. In the present work, we have considered a spherically confined hydrogenic atom, i.e., the hydrogen atom (H), the helium-ion ( He$$^{+1})$$, or the lithium-ion (Li$$^{+2})$$, in PTDRSC potential. The PTDRSC potential reduces to Coulomb potential or double ring-shaped Coulombic potential for a few typical values of parameters. We have considered complete wave functions since PTDRSC potential affects the angular and azimuthal solutions. We solved the time-independent Schrödinger equation in MATLAB using the 9-point finite difference method to estimate energy eigenvalues and complete wave functions. We model a spherically unbounded system by choosing a large-size spherical boundary, $$r_0 = 50$$ a.u. The energy spectrum corresponding to this spherical boundary matches that of a free atom. In the present work, we have considered a few sets of the PTDRSC potential parameters i.e., $$\alpha , c, d $$ and $$\beta , $$ and *a*. Energies of various states of H, He$$^{+1}$$, and Li$$^{+2}$$ for some typical combination of parameters of PTDRSC potential when $$r_0=50$$ a.u. are enlisted in Table 1. This composite potential captures the combined effects of these three components, resulting in a unique and complex energy landscape. To understand the effect of atomic number *Z* on the energy spectrum, we have also enlisted energy values for He$$^{+1}$$ and Li$$^{+2}$$. The presence of PTDRSC potential modifies the energy spectrum of the hydrogenic atom. Theoretical energy values estimated using the analytical solution available for the energy spectrum in PTDRSC potential^34^. It can be observed from Table 1 that there is a consistent match between the theoretically estimated and our numerically evaluated energy levels for all the combinations of the parameter of PTDRSC potential. A few values of modified magnetic quantum number, $$m'$$, have been enlisted in the last three columns of Table 1. Angular distortion, i.e., modification in the angular quantum number, $$l'$$, is introduced due to angular dependence of PTDRSC potential. PTDRSC potential modifies magnetic quantum number, $$m'$$, due to azimuthal dependence, frequently referred to as azimuthal distortion. The angular dependence of PTDRSC potential does not influence azimuthal distortion. In contrast, the azimuthal dependence of PTDRSC potential does affect angular distortion, as can be clearly observed from Table 1. The energy spectrum of the system is linked to parameters of PTDRSC potential and three quantum numbers.

The wavefunction, in turn, affects the current density, which further modifies the persistent current, *I*, and its corresponding induced magnetic field, *B*. To visualize the effect of PTDRSC potential on the motion of electron associated with the hydrogenic atom, we have estimated persistent current for a few states of the system. We have evaluated the magnitude of persistent current in different states and the corresponding induced magnetic field at the nucleus of the hydrogen atom (H), the helium-ion (He$$^{+1})$$, and the lithium-ion (Li$$^{+2})$$. We estimate *I* and *B* at the nucleus ($$r=0$$) for states characterized by three quantum numbers, i.e., $$n, l',$$ and $$m'$$. Solving the time-independent Schrödinger equation for the hydrogen atom (*H*) with $$Z=1$$. We can easily estimate solutions for arbitrary *Z* using effective interaction. Our results for the persistent current and its corresponding induced magnetic field at the nucleus of the above-considered hydrogenic atoms in $$2p{\pm 1}$$ state closely match with the available results^24^. Table 2 enlists the value of the same in a few states, i.e., 1*s*0, 2*s*0, $$2p{\pm 1}$$, and 2*p*0 for the hydrogenic atoms. Among these states, there is a persistent current only in the $$2p{\pm 1}$$ state of the hydrogenic atoms. The same story follows even when the potential has angular dependence. The persistent current for the $$2p{\pm 1}$$ state decreases in the presence of double ring-shaped angular potential as observable from the first block of Table 2 corresponding to $$\alpha =1, c=1, d=1 $$ and different combinations of $$\beta , $$ and *a*. Surprisingly, as the potential starts having azimuthal dependence, induced current is observed in states corresponding to $$m=0$$, as enlisted in the second block corresponding to $$\beta =0,$$ $$a=1.0$$ and different combinations of $$\alpha , c, d $$. Interestingly, we observe a large current in states corresponding to $$m=0$$ (notably, there is a persistent current for S-states). The induced current amplitude in 1*s*0 and 2*p*0 state is more than that for $$2p{\pm 1}$$ state, and the amplitude of this current depends on both angular and azimuthal potential parameters of the PTDRSC potential. The persistent current can be fine-tuned by controlling the strength of these parameters. A current of the order of 5*mA* is observed in 1*s*0 state of Li$$^{+2}$$ with a particular combination of PTDRSC potential parameters (i.e., $$\alpha =0.1, c=d=1.01, \beta =0$$, $$a=1.0$$). A similar approach is to explore the induced magnetic field at the nucleus of the above-considered hydrogenic atoms. Table 3 gives the amplitude of the induced magnetic field for a few states, i.e., 1*s*0, 2*s*0, $$2p{\pm 1}$$, and 2*p*0 at the nucleus of the hydrogenic atom. We observe a significantly large induced magnetic field corresponding to different S-states. Similar to the persistent current, the amplitude of the induced magnetic field varies with both the angular and azimuthal potential parameters of the PTDRSC potential. A significantly high magnetic field of 52*T* is estimated for a certain combination of parameters of PTDRSC potential (i.e., $$\alpha =0.1, c=d=1.01, \beta =0$$, $$a=1.0$$).

**Figure 1:**
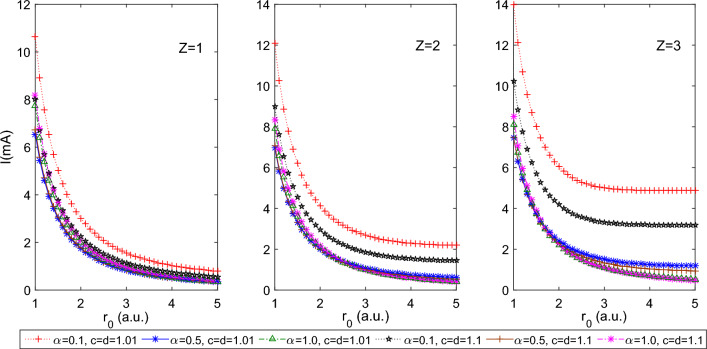
Plot of the persistent current of 1s0 state for the hydrogenic atoms (H, He$^{+1}$ and Li$^{+2}$, i.e., Z = 1, 2, and 3 respectively) for a few combinations of azimuthal potential parameters (i.e., $\alpha , c,$ and *d*) with $r_{0}$.

**Figure 2:**
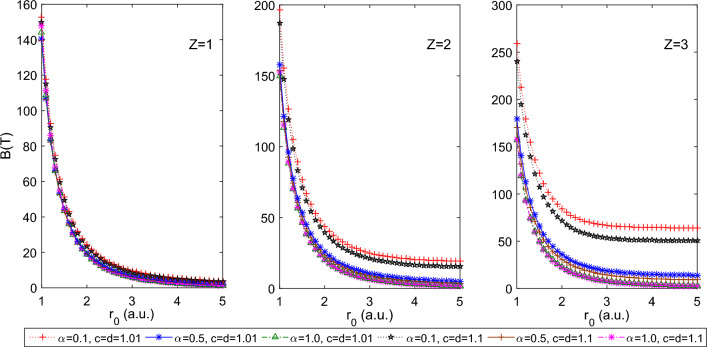
Plot of the induced magnetic field of 1s0 state at the nucleus for the hydrogenic atoms (H, He$^{+1}$ and Li$^{+2}$, i.e., Z = 1, 2, and 3 respectively) for a few combinations of azimuthal potential parameters (i.e., $\alpha , c,$ and *d*) with $r_{0}$.

**Figure 3:**
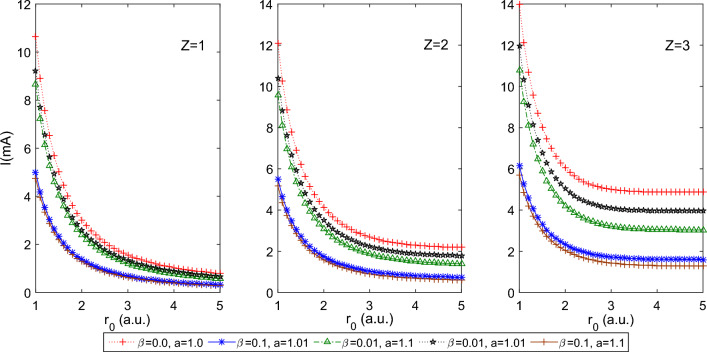
Plot of the persistent current of 1s0 state for the hydrogenic atoms (H, He$^{+1}$ and Li$^{+2}$, i.e., Z = 1, 2, and 3 respectively) for a few combinations of angular potential parameters (i.e., $\beta$ and *a*) for constant azimuthal potential parameters (i.e., $\alpha =0.1, c = d = 1.01$) with $r_{0}$.

**Figure 4:**
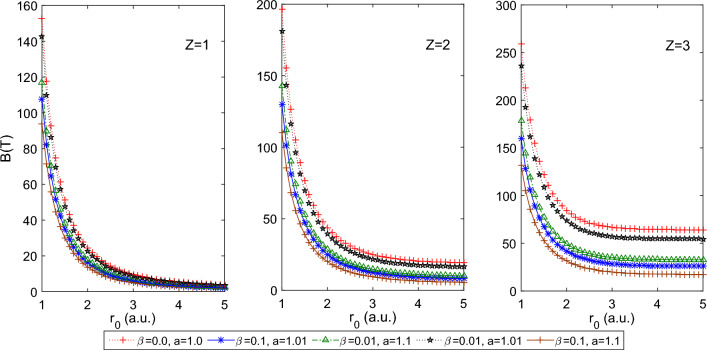
Plot of the induced magnetic field of 1s0 state at the nucleus for the hydrogenic atoms (H, He$^{+1}$ and Li$^{+2}$, i.e., Z = 1, 2, and 3 respectively) for a few combinations of angular potential parameters (i.e., $\beta$ and *a*) for constant azimuthal potential parameters (i.e., $\alpha =0.1, c = d = 1.01$) with $r_{0}$.

The wave function of a confined system depends strongly on the type and strength of confinement potential. The present work focuses on estimating persistent currents and their corresponding induced magnetic fields in different states of the spherically confined hydrogenic atoms in PTDRSC potential. Furthermore, we explore how the size of the spherical boundary contributes to the variations in the persistent current and induced magnetic field of different states. Figure 1 shows the variation of persistent current for 1*s*0 state of the hydrogenic atoms (i.e., for $Z=1, 2,$ and 3) with a few combinations of azimuthal potential parameters of PTDRSC potential, i.e., $\alpha , c$, and *d*. The angular potential parameters were considered as $\beta =0,$ $a=1.0$, which reduces PTDRSC potential to have only azimuthal dependence. As seen from Fig. 1, a decrease in the size of the spherical boundary, i.e., tightly confined system, leads to a monotonic increase in persistent current, *I*. There is a prominent increase in the persistent current with *Z* (i.e., for $Z = 1, 2,$ and 3), whereas, under tight confinement, the effect of *Z* is diminished. Figure 2 shows the corresponding variation of the induced magnetic field of 1*s*0 state at the nucleus of the hydrogenic atom with $r_0$. The significant magnetic field is induced for several combinations of parameters of PTDRSC potential under tight confinement. To understand the influence of angular potential parameters, we have considered a few combinations of angular potential parameters while keeping the azimuthal potential parameters constant, i.e., $\alpha = 0.1, c = d = 1.01$. It can be seen from Fig. 3 that hydrogenic atoms exhibit a variation of persistent current with the choice of angular potential parameters. Again, with a reduction in the size of the spherical boundary, the persistent current rises. So, the amplitude of persistent current can be adjusted by varying potential parameters and the size of the spherical boundary. Figure 4 shows the corresponding variation of the induced magnetic field of 1*s*0 state at the nucleus of the hydrogenic atom with $r_0$. A strong induced magnetic field of approximately 250*T* can be generated by tuning the parameters of PTDRSC potential and size of the spherical boundary.

**Figure 5:**
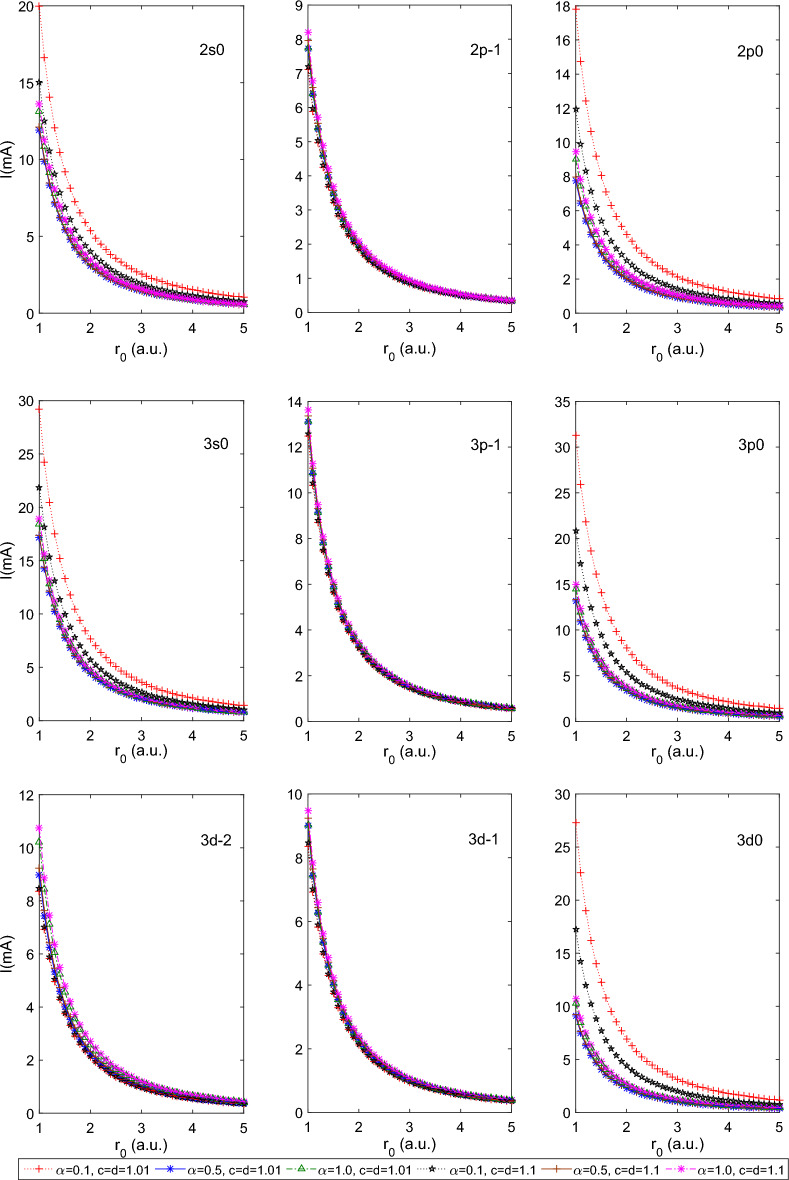
Plot of the persistent current of a few states of the hydrogen atom for a few combinations of azimuthal potential parameters (i.e., $\alpha , c,$ and *d*) with $r_{0}$.

**Figure 6:**
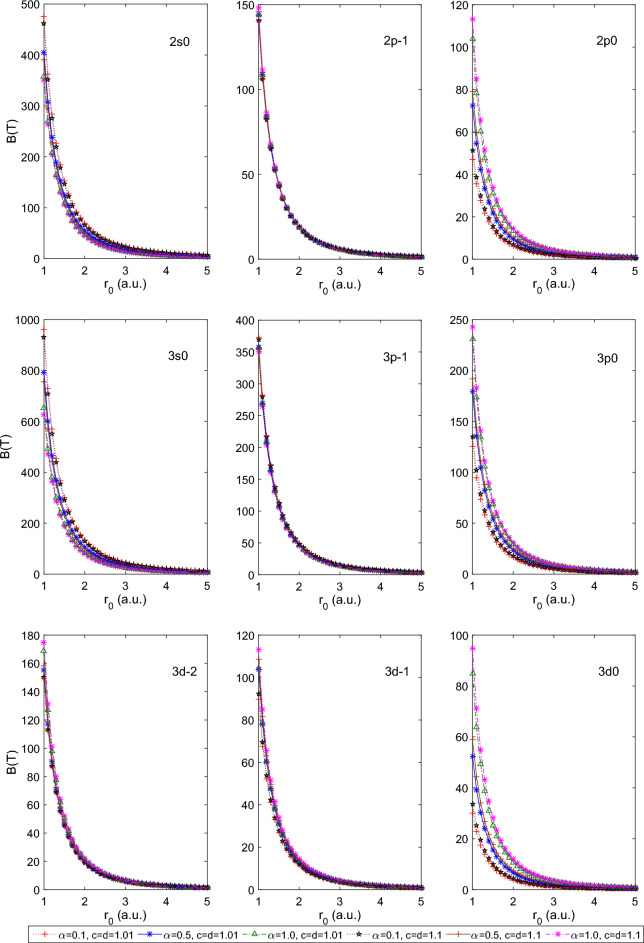
Plot of the induced magnetic field of a few states of the hydrogen atom for a few combinations of azimuthal potential parameters (i.e., $\alpha , c,$ and *d*) with $r_{0}$.

**Figure 7:**
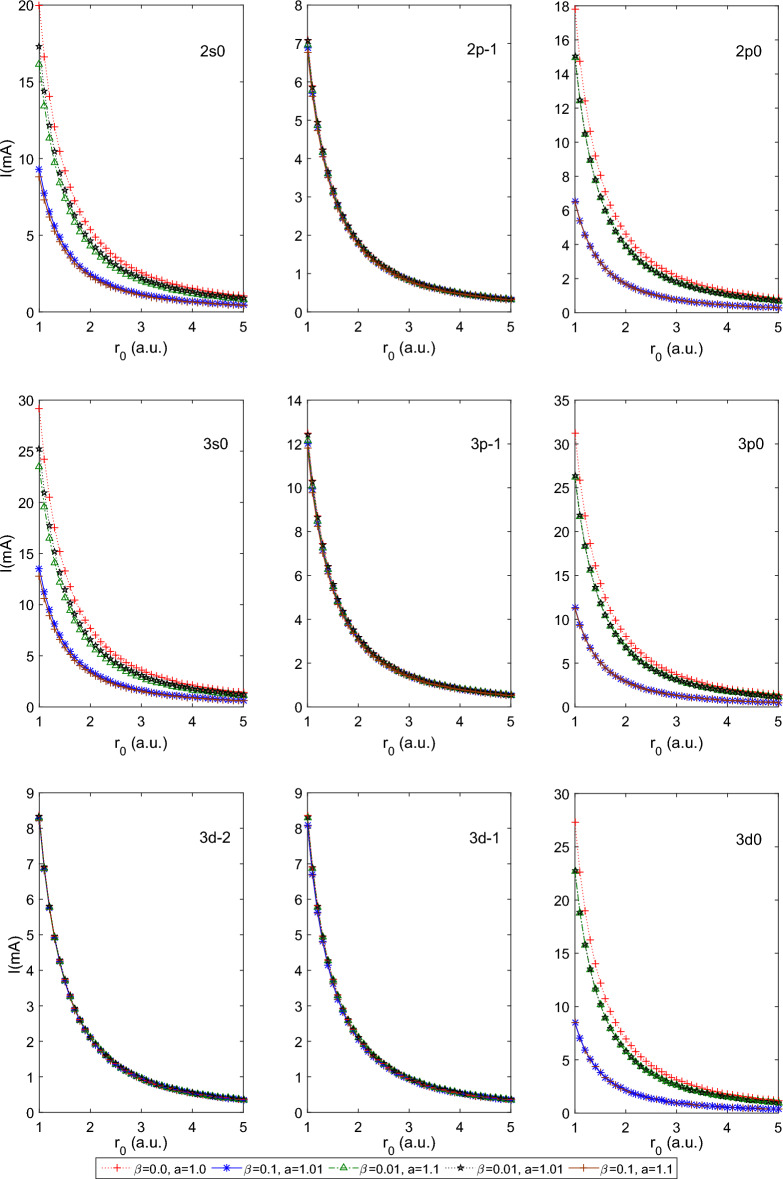
Plot of the persistent current of a few states of the hydrogen atom for a few combinations of angular potential parameters (i.e., $\beta$ and *a*) for constant azimuthal potential parameters (i.e., $\alpha =0.1, c = d = 1.01$) with $r_{0}$.

**Figure 8:**
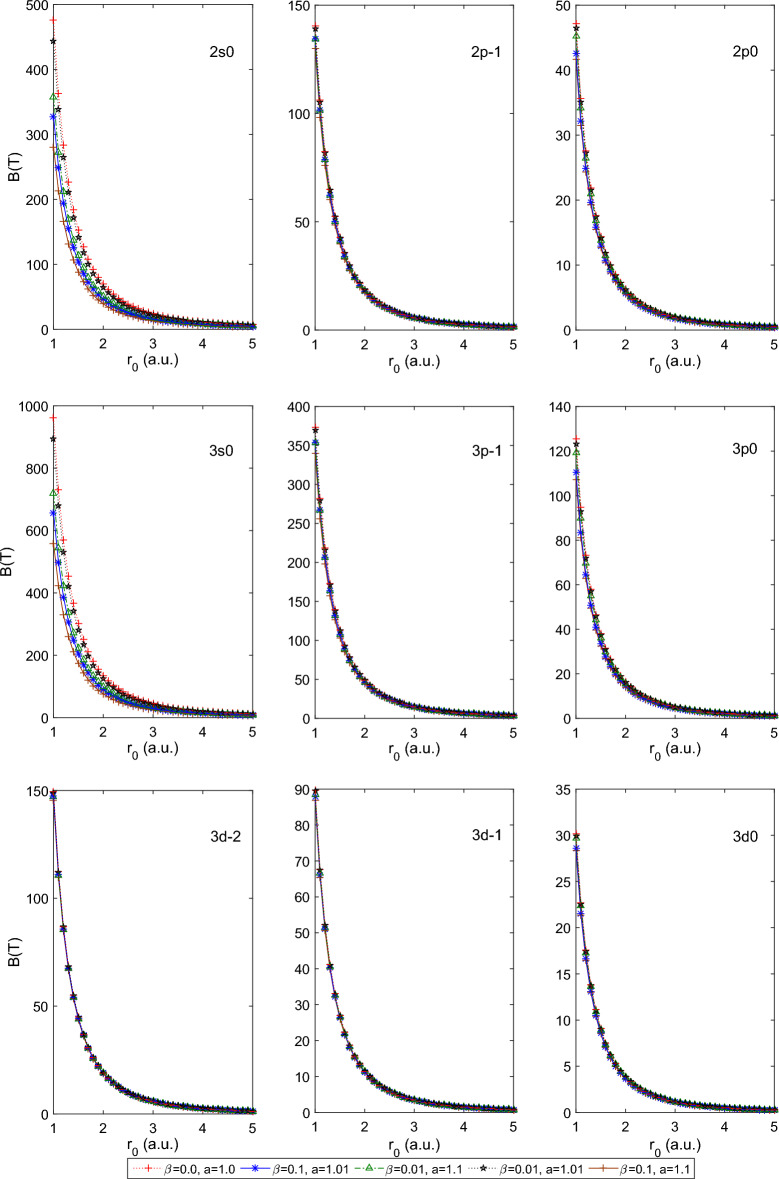
Plot of the induced magnetic field of a few states of the hydrogen atom for a few combinations of angular dependent potential parameters (i.e., $\beta$ and *a*) for constant azimuthal potential parameters (i.e., $\alpha =0.1, c = d = 1.01$) with $r_{0}$.

Let us explore the impact of PTDRSC potential parameters and the size of the spherical boundary on the persistent current of other states i.e., $2s0, 2p{\pm 1}, 2p0, 3s0, 3p{\pm 1}, 3p0, 3d{\pm 2}, 3d{\pm 1},$ and 3*d*0. The variation of persistent current of these states of the hydrogen atom with the size of the boundary, $r_0$, is shown in Fig. 5. We have considered a few combinations of azimuthal potential parameters (i.e., $\alpha , c,$ and *d*) while keeping the angular potential parameters as $\beta =0,$ $a=1.0$. This combination of parameters reduces the PTDRSC potential to have only azimuthal dependence. After carefully analyzing the variation patterns, we can conclude that the states corresponding to $m=0$ (i.e., 2*s*0, 3*s*0,  and 3*d*0) are very sensitive to azimuthal potential parameters, i.e., $\alpha , c,$ and *d*. Figure 6 shows a beautiful variation of the induced magnetic field of the above-mentioned states of the hydrogen atom. A very high induced magnetic field of 1000*T* is estimated for the 3*s*0 state. Let us consider a few combinations of angular potential parameters (i.e., $\beta ,$ and *a*) while keeping the azimuthal potential parameters as $\alpha =0.1, c=d=1.01$ to explore the induced magnetic field thoroughly. Figure 7 shows the variation of the persistent current of the above-considered states of the hydrogen atom. A significantly high current (around 30*mA*) is observed for the 3*p*0 state at $r_0=1$ a.u. It is clearly observable that the states corresponding to $m=0$ show sensitivity to angular potential parameters. Similarly, Fig. 8 depicts the variation of the induced magnetic field of these states of the hydrogen atom.

**Table 1:** Variation of the energy of 1*s*0 and 2*s*0 states of the hydrogenic atoms, i.e., hydrogen atom (H), helium ion (He$$^{+1})$$ and lithium-ion (Li$$^{+2})$$ for a few combination of parameters of Pöschl–Teller double-ring-shaped Coulomb (PTDRSC) potential when $$r_0=50$$ a.u.

	H				He$^{+1}$		Li$^{+2}$		$\Lambda$		
Parameters	$E_{th}(1s0)$	$E_{cal}(1s0)$	$E_{th}(2s0)$	$E_{cal}(2s0)$	*E*(1*s*0)	*E*(2*s*0)	*E*(1*s*0)	*E*(2*s*0)	$m=0$	$m=-1$	$m=-2$
$$\alpha =1, c=d=1$$											
$\beta =0$, $a=1$	− 0.50000	− 0.50000	− 0.12500	− 0.12500	− 1.99954	− 0.50000	− 4.48219	− 1.12482	0.00000	− 1.00000	− 2.00000
$\beta =0.01$, $a=1$	− 0.38378	− 0.40842	− 0.10903	− 0.11270	− 1.66835	− 0.45578	− 3.83439	− 1.03754	0.00000	− 1.00000	− 2.00000
$\beta =0.1$, $a=1$	− 0.23873	− 0.24009	− 0.08349	− 0.08379	− 0.97394	− 0.33810	− 2.24826	− 0.77272	0.00000	− 1.00000	− 2.00000
$\beta =0$, $a=1.01$	− 0.48077	− 0.48252	− 0.12256	− 0.12279	− 1.93921	− 0.49234	− 4.36893	− 1.11013	0.00000	− 1.00000	− 2.00000
$\beta =0$, $a=1.1$	− 0.35573	− 0.35810	− 0.10467	− 0.10507	− 1.47015	− 0.42631	− 3.41316	− 0.97570	0.00000	− 1.00000	− 2.00000
$\beta =0.01$, $a=1.01$	− 0.37080	− 0.39637	− 0.10705	− 0.11094	− 1.62191	− 0.44911	− 3.73811	− 1.02381	0.00000	− 1.00000	− 2.00000
$\beta =0.01$, $a=1.1$	− 0.28395	− 0.30832	− 0.09234	− 0.09676	− 1.26417	− 0.39259	− 2.94652	− 0.90101	0.00000	− 1.00000	− 2.00000
$\beta =0.1$, $a=1.01$	− 0.23233	− 0.23618	− 0.08215	− 0.08297	− 0.95730	− 0.33468	− 2.20752	− 0.76446	0.00000	− 1.00000	− 2.00000
$\beta =0.1$, $a=1.1$	− 0.18755	− 0.20410	− 0.07213	− 0.07599	− 0.82223	− 0.30541	− 1.87786	− 0.69385	0.00000	− 1.00000	− 2.00000
$$\beta =0, a=1.0$$											
$\alpha =0.1$, c = d = 1.01	− 0.34494	− 0.32724	− 0.10293	− 0.10002	− 1.34340	− 0.40598	− 3.12943	− 0.93117	− 0.50537	− 1.50139	− 2.49611
$\alpha =0.5$, c = d = 1.01	− 0.12256	− 0.12474	− 0.05483	− 0.05548	− 0.49897	− 0.22192	− 1.12268	− 0.49931	− 1.00937	− 1.99851	− 2.98742
$\alpha =1.0$, c = d = 1.01	− 0.05412	− 0.05608	− 0.03064	− 0.03146	− 0.22433	− 0.12589	− 0.50474	− 0.28325	− 2.00411	− 2.01815	− 3.96717
$\alpha =0.1$, c = d = 1.1	− 0.32670	− 0.30573	− 0.09991	− 0.09631	− 1.25327	− 0.39071	− 2.92105	− 0.89671	− 0.57381	− 1.58648	− 2.58885
$\alpha =0.5$, c = d = 1.1	− 0.10467	− 0.10470	− 0.04927	− 0.04928	− 0.41876	− 0.19710	− 0.94195	− 0.44338	− 1.18545	− 2.18529	− 3.18511
$\alpha =1.0$, c = d = 1.1	− 0.04400	− 0.04402	− 0.02617	− 0.02608	− 0.17609	− 0.10472	− 0.39619	− 0.23562	− 2.37066	− 2.37084	− 4.37003
$\beta =0.1$, a=1.01											
$\alpha =0.1$, c = d = 1.01	− 0.21890	− 0.21568	− 0.07928	− 0.07858	− 0.87070	− 0.31622	− 1.99567	− 0.71990	− 0.50537	− 1.50139	− 2.49611
$\alpha =0.5$, c = d = 1.01	− 0.10986	− 0.11288	− 0.05093	− 0.05187	− 0.45147	− 0.20748	− 1.01556	− 0.46673	− 1.00937	− 1.99851	− 2.98742
$\alpha =1.0$, c = d = 1.01	− 0.05177	− 0.05409	− 0.02963	− 0.03061	− 0.21635	− 0.12251	− 0.48679	− 0.27565	− 2.00411	− 2.01815	− 3.96717
$\beta =0.01$, a=1.1											
$\alpha =0.1$, c = d = 1.01	− 0.24323	− 0.24815	− 0.08441	− 0.08543	− 1.00823	− 0.34505	− 2.33222	− 0.78943	− 0.50537	− 1.50139	− 2.49611
$\alpha =0.5$, c = d = 1.01	− 0.10190	− 0.11406	− 0.04837	− 0.05224	− 0.45619	− 0.20895	− 1.02619	− 0.47004	− 1.00937	− 1.99851	− 2.98742
$\alpha =1.0$, c = d = 1.01	− 0.04792	− 0.05397	− 0.02794	− 0.03055	− 0.21586	− 0.12230	− 0.48570	− 0.27518	− 2.00411	− 2.01815	− 3.96717
$\beta =0.01$, a=1.01											
$\alpha =0.1$, c = d = 1.01	− 0.31098	− 0.29995	− 0.09720	− 0.09528	− 1.22882	− 0.38643	− 2.86363	− 0.88692	− 0.50537	− 1.50139	− 2.49611
$\alpha =0.5$, c = d = 1.01	− 0.11905	− 0.12257	− 0.05377	− 0.05483	− 0.49028	− 0.21933	− 1.10307	− 0.49346	− 1.00937	− 1.99851	− 2.98742
$\alpha =1.0$, c = d = 1.01	− 0.05325	− 0.05571	− 0.03027	− 0.03130	− 0.22284	− 0.12526	− 0.50140	− 0.28184	− 2.00411	− 2.01815	− 3.96717
$\beta =0.1$, a=1.1											
$\alpha =0.1$, c = d = 1.01	− 0.17777	− 0.18900	− 0.06977	− 0.07248	− 0.75954	− 0.29086	− 1.72694	− 0.65900	− 0.50537	− 1.50139	− 2.49611
$\alpha =0.5$, c = d = 1.01	− 0.09459	− 0.10566	− 0.04594	− 0.04959	− 0.42261	− 0.19834	− 0.95062	− 0.44618	− 1.00937	− 1.99851	− 2.98742
$\alpha =1.0$, c = d = 1.01	− 0.04666	− 0.05244	− 0.02738	− 0.02990	− 0.20977	− 0.11969	− 0.47199	− 0.26931	− 2.00411	− 2.01815	− 3.96717

**Table 2:** Variation of the persistent current of few states for the hydrogenic atoms, i.e., hydrogen atom (H), helium ion (He$$^{+1})$$ and lithium-ion (Li$$^{+2})$$ for a few combination of parameters of Pöschl–Teller double-ring-shaped Coulomb (PTDRSC) potential when $$r_0=50$$ a.u.

	H				He$^{+1}$				Li$^{+2}$			
Parameters	I(1s0)	I(2s0)	I(2p-1)	I(2p0)	I(1s0)	I(2s0)	I(2p-1)	I(2p0)	I(1s0)	I(2s0)	I(2p-1)	I(2p0)
$\alpha =1$, c = d = 1												
$\beta =0$, $a=1$	0.00000	0.00000	0.13194	0.00000	0.00000	0.00000	0.52824	0.00000	0.00000	0.00000	1.19299	0.00000
$\beta =0.01$, $a=1$	0.00000	0.00000	0.12869	0.00000	0.00000	0.00000	0.51513	0.00000	0.00000	0.00000	1.16280	0.00000
$\beta =0.1$, $a=1$	0.00000	0.00000	0.10462	0.00000	0.00000	0.00000	0.41837	0.00000	0.00000	0.00000	0.94156	0.00000
$\beta =0$, $a=1.01$	0.00000	0.00000	0.12981	0.00000	0.00000	0.00000	0.51965	0.00000	0.00000	0.00000	1.17314	0.00000
$\beta =0$, $a=1.1$	0.00000	0.00000	0.11145	0.00000	0.00000	0.00000	0.44573	0.00000	0.00000	0.00000	1.00347	0.00000
$\beta =0.01$, $a=1.01$	0.00000	0.00000	0.12663	0.00000	0.00000	0.00000	0.50685	0.00000	0.00000	0.00000	1.14372	0.00000
$\beta =0.01$, $a=1.1$	0.00000	0.00000	0.10890	0.00000	0.00000	0.00000	0.43550	0.00000	0.00000	0.00000	0.98020	0.00000
$\beta =0.1$, $a=1.01$	0.00000	0.00000	0.10310	0.00000	0.00000	0.00000	0.41228	0.00000	0.00000	0.00000	0.92769	0.00000
$\beta =0.1$, $a=1.1$	0.00000	0.00000	0.08982	0.00000	0.00000	0.00000	0.35908	0.00000	0.00000	0.00000	0.80704	0.00000
$\beta =0$, a=1.0												
$\alpha =0.1$, c = d = 1.01	0.55055	0.11928	0.06735	0.16799	2.29575	0.49464	0.26935	0.67180	5.43701	1.15502	0.60542	1.51005
$\alpha =0.5$, c = d = 1.01	0.13008	0.03872	0.03910	0.03880	0.52071	0.15503	0.15641	0.15521	1.17545	0.35011	0.35190	0.34922
$\alpha =1.0$, c = d = 1.01	0.03888	0.01652	0.03834	0.01645	0.15553	0.06568	0.15337	0.06568	0.34994	0.14778	0.34508	0.14779
$\alpha =0.1$, c = d = 1.1	0.35790	0.08179	0.06092	0.10263	1.48095	0.33753	0.24365	0.41046	3.50360	0.79154	0.54784	0.92284
$\alpha =0.5$, c = d = 1.1	0.10100	0.03261	0.03262	0.03263	0.40373	0.13037	0.13048	0.13050	0.90729	0.29294	0.29357	0.29364
$\alpha =1.0$, c = d = 1.1	0.02753	0.01304	0.02752	0.01275	0.11011	0.05050	0.11009	0.05050	0.24775	0.11364	0.24771	0.11364
$\beta =0.1$, a = 1.01												
$\alpha =0.1$, c = d = 1.01	0.17841	0.04408	0.05920	0.04890	0.72822	0.17988	0.23677	0.19559	1.70083	0.41891	0.53233	0.43990
$\alpha =0.5$, c = d = 1.01	0.10202	0.03188	0.03610	0.03211	0.40792	0.12746	0.14439	0.12846	0.91770	0.28679	0.32488	0.28903
$\alpha =1.0$, c = d = 1.01	0.03591	0.01549	0.03545	0.01544	0.14364	0.06147	0.14178	0.06163	0.32319	0.13831	0.31900	0.13866
$\beta$=0.01, a=1.1												
$\alpha =0.1$, c = d = 1.01	0.33900	0.08112	0.05996	0.12921	1.39149	0.33261	0.23982	0.51674	3.27080	0.77797	0.53918	1.16179
$\alpha =0.5$, c = d = 1.01	0.10759	0.03346	0.03615	0.03615	0.43023	0.13378	0.14459	0.14459	0.96814	0.30109	0.32532	0.32532
$\alpha =1.0$, c = d = 1.01	0.03596	0.01553	0.03549	0.01587	0.14383	0.06160	0.14194	0.06335	0.32360	0.13860	0.31936	0.14253
$\beta$=0.01, a=1.01												
$\alpha =0.1$, c = d = 1.01	0.44656	0.09911	0.06588	0.13745	1.85627	0.41030	0.26344	0.54968	4.39856	0.96097	0.59218	1.23566
$\alpha =0.5$, c = d = 1.01	0.12492	0.03751	0.03855	0.03786	0.49993	0.15013	0.15421	0.15143	1.12765	0.33875	0.34696	0.34072
$\alpha =1.0$, c = d = 1.01	0.03834	0.01633	0.03781	0.01630	0.15336	0.06492	0.15125	0.06510	0.34505	0.14607	0.34031	0.14646
$\beta$=0.1, a=1.1												
$\alpha =0.1$, c = d = 1.01	0.14238	0.03728	0.05413	0.04619	0.57600	0.15088	0.21650	0.18474	1.32806	0.34772	0.48684	0.41553
$\alpha =0.5$, c = d = 1.01	0.08898	0.02869	0.03391	0.03073	0.35572	0.11468	0.13565	0.12294	0.79942	0.25770	0.30520	0.27661
$\alpha =1.0$, c = d = 1.01	0.03374	0.01475	0.03333	0.01504	0.13497	0.05841	0.13330	0.06001	0.30368	0.13143	0.29992	0.13501

**Table 3:** Variation of the induced magnetic field at the nucleus for the hydrogenic atoms, i.e., hydrogen atom (H), helium ion (He$$^{+1})$$ and lithium-ion (Li$$^{+2})$$ for a few combination of parameters of Pöschl–Teller double-ring-shaped Coulomb (PTDRSC) potential when $$r_0=50$$ a.u.

Parameters	H				He$^{+1}$				Li$^{+2}$			
	B(1s0)	B(2s0)	B(2p-1)	B(2p0)	B(1s0)	B(2s0)	B(2p-1)	B(2p0)	B(1s0)	B(2s0)	B(2p-1)	B(2p0)
$\alpha$=1, c = d = 1												
$\beta =0$, $a=1$	0.00000	0.00000	0.52150	0.00000	0.00000	0.00000	4.16835	0.00000	0.00000	0.00000	14.02362	0.00000
$\beta =0.01$, $a=1$	0.00000	0.00000	0.50289	0.00000	0.00000	0.00000	4.02009	0.00000	0.00000	0.00000	13.52713	0.00000
$\beta =0.1$, $a=1$	0.00000	0.00000	0.37149	0.00000	0.00000	0.00000	2.97141	0.00000	0.00000	0.00000	10.00926	0.00000
$\beta =0$, $a=1.01$	0.00000	0.00000	0.50694	0.00000	0.00000	0.00000	4.05233	0.00000	0.00000	0.00000	13.63510	0.00000
$\beta =0$, $a=1.1$	0.00000	0.00000	0.38879	0.00000	0.00000	0.00000	3.10958	0.00000	0.00000	0.00000	10.47335	0.00000
$\beta =0.01$, $a=1.01$	0.00000	0.00000	0.48902	0.00000	0.00000	0.00000	3.90952	0.00000	0.00000	0.00000	13.15668	0.00000
$\beta =0.01$, $a=1.1$	0.00000	0.00000	0.37621	0.00000	0.00000	0.00000	3.00907	0.00000	0.00000	0.00000	10.13577	0.00000
$\beta =0.1$, $a=1.01$	0.00000	0.00000	0.36224	0.00000	0.00000	0.00000	2.89742	0.00000	0.00000	0.00000	9.76072	0.00000
$\beta =0.1$, $a=1.1$	0.00000	0.00000	0.28557	0.00000	0.00000	0.00000	2.28445	0.00000	0.00000	0.00000	7.69974	0.00000
$\beta$=0, a=1.0												
$\alpha$=0.1, c = d = 1.01	2.25830	0.48540	0.15974	0.05328	16.84951	3.55772	1.27764	0.42614	52.77206	10.77255	4.30948	1.43736
$\alpha$=0.5, c = d = 1.01	0.50868	0.15143	0.06197	0.03055	4.06633	1.21033	0.49574	0.24439	13.68259	4.06923	1.67280	0.82466
$\alpha$=1.0, c = d = 1.01	0.06137	0.02610	0.05990	0.00929	0.49095	0.20733	0.47920	0.07416	1.65664	0.69954	1.61701	0.25030
$\alpha$=0.1, c = d = 1.1	1.82530	0.41497	0.13404	0.05018	13.96212	3.13618	1.07206	0.40136	44.93777	9.85199	3.61627	1.35385
$\alpha$=0.5, c = d = 1.1	0.32556	0.10514	0.04528	0.02455	2.60436	0.84112	0.36223	0.19643	8.77856	2.83535	1.22243	0.66291
$\alpha$=1.0, c = d = 1.1	0.03378	0.01609	0.03377	0.00631	0.27023	0.12395	0.27016	0.04986	0.91201	0.41831	0.91177	0.16828
$\beta$=0.1, a=1.01												
$\alpha$=0.1, c = d = 1.01	0.95531	0.23548	0.13170	0.03358	7.48766	1.83504	1.05337	0.26856	24.71227	5.97876	3.55320	0.90597
$\alpha$=0.5, c = d = 1.01	0.35511	0.11097	0.05497	0.02465	2.84051	0.88770	0.43971	0.19719	9.56971	2.99028	1.48378	0.66544
$\alpha$=1.0, c = d = 1.01	0.05447	0.02352	0.05324	0.00855	0.43571	0.18646	0.42588	0.06821	1.47028	0.62916	1.43712	0.23020
$\beta$=0.01, a=1.1												
$\alpha$=0.1, c = d = 1.01	1.18666	0.28299	0.13182	0.04450	9.22275	2.18181	1.05432	0.35591	30.18282	7.01916	3.55642	1.20056
$\alpha$=0.5, c = d = 1.01	0.36832	0.11454	0.05455	0.02738	2.94607	0.91619	0.43638	0.21901	9.92440	3.08583	1.47253	0.73905
$\alpha$=1.0, c = d = 1.01	0.05405	0.02337	0.05283	0.00877	0.43239	0.18519	0.42259	0.06998	1.45908	0.62489	1.42603	0.23618
$\beta$=0.01, a=1.01												
$\alpha$=0.1, c = d = 1.01	1.94000	0.42773	0.15428	0.04990	14.65176	3.18214	1.23399	0.39908	46.51023	9.80907	4.16228	1.34613
$\alpha$=0.5, c = d = 1.01	0.47742	0.14335	0.06062	0.02965	3.81707	1.14606	0.48493	0.23715	12.84736	3.85485	1.63634	0.80024
$\alpha$=1.0, c = d = 1.01	0.06004	0.02560	0.05862	0.00917	0.48029	0.20333	0.46891	0.07323	1.62069	0.68604	1.58231	0.24714
$\beta$=0.1, a=1.1												
$\alpha$=0.1, c = d = 1.01	0.63937	0.16724	0.11342	0.03033	5.06487	1.32109	0.90720	0.24260	16.88702	4.37396	3.06026	0.81843
$\alpha$=0.5, c = d = 1.01	0.28059	0.09047	0.04963	0.02286	2.24459	0.72377	0.39700	0.18289	7.56575	2.43971	− 1.33970	0.61720
$\alpha$=1.0, c = d = 1.01	0.04919	0.02154	0.04813	0.00818	0.39354	0.17031	0.38504	0.06524	1.32803	0.57470	1.29937	0.22020

## Conclusion

In this work, we estimate the influence of PTDRSC potential parameters on the energy spectrum of hydrogenic atoms. We observed persistent current in S-states of the confined hydrogenic atoms (H, He$$^{+1}$$, Li$$^{+2})$$ under the influence of Pöschl-Teller double-ring-shaped Coulomb (PTDRSC) potential. PTDRSC potential affects the magnetic quantum number ($$m'$$), which corresponds to a significant persistent current in states corresponding to $$m=0$$. In free hydrogenic atoms, persistent current corresponding to $$m=0$$ states is significantly greater than other states. The magnitude of *I* and *B* at the nucleus depends on three quantum numbers ($$n, l'$$ and $$m'$$). This investigation examines the effect of different parameters of PTDRSC potential, i.e., angular and azimuthal potential parameters and the size of the spherical boundary on persistent currents and the corresponding induced magnetic fields in different states. The PTDRSC potential strongly affects the persistent current and its corresponding induced magnetic field. Our results showcase the possibility of manipulating both quantities in the S-state by tuning the strength of PTDRSC potential. This research has potential implications for fundamental physics, quantum computing and information processing applications.

## Data Availability

The datasets generated during and/or analysed during the current study are available from the corresponding author upon reasonable request.
